# The use of planned behavior theory in predicting cigarette smoking among Waterpipe smokers

**DOI:** 10.1186/s12971-017-0133-z

**Published:** 2017-07-03

**Authors:** Naif H. Alanazi, Jerry W. Lee, Hildemar Dos Santos, Jayakaran S. Job, Khaled Bahjri

**Affiliations:** 1 Loma Linda University, Loma Linda, California USA; 2 Health Promotion and Education, Loma Linda University, Loma Linda, California USA; 3 Epidemiology/Biostatistics/Population Medicine, Schools of Public Health and Medicine, Loma Linda, California USA; 4 Epidemiology, Biostatistics, and Population Medicine, Loma Linda, California USA

**Keywords:** Theory of planned behavior predicts cigarette use, Waterpipe, Hookah, & cigarette smoking

## Abstract

**Background:**

Waterpipe and cigarette smoking have been found to be associated with each other as cigarette smokers were more likely to be waterpipe users than non-cigarette smokers. Also, waterpipe smokers were likely to be former daily cigarette users. The aim of this study is to examine the likelihood of waterpipe use leading to cigarette use among current waterpipe users using theory of planned behavior.

**Methods:**

Four hundred six current waterpipe smokers who initially had started tobacco use with the waterpipe were recruited from 15 waterpipe lounges in 2015. From a total of 70 waterpipe lounges in Riyadh, the 15 waterpipe lounges were selected randomly and participants were also selected randomly inside each waterpipe lounge based on the table or section number. The survey was developed using the Qualtrics Online Survey Software and participants completed a survey using iPad tablets.

**Results:**

Cigarette smoking and intention to smoke cigarettes were predicted by attitude and perceived behavioral control. There was no direct effect of subjective norm on the cigarette use behavior, yet subjective norm had a statistically significant indirect effect on intentions through attitude and perceived behavioral control.

**Conclusions:**

The findings of this study could be useful in prevention/intervention programs aimed at reducing tobacco smoking behaviors among waterpipe users. Intervention programs might be directed at the attitude and perceived behavioral control by targeting underlying behavioral and control beliefs. The theory of planned behavior provided solid explanations of intention to use cigarettes among waterpipe smokers.

## Background

The prevalence of waterpipe smoking in the Middle East appears to be very high, especially among students and young adults [1]. One study conducted in some colleges of the Qassim University, Saudi Arabia revealed that total prevalence of waterpipe use was 40% [2]. Additionally, a study from four Jordanian universities showed that current waterpipe use prevalence was 30% among university students and 56% had or were continuing to use waterpipe [3]. While waterpipe continues to be popular in the Middle East, it is becoming increasingly popular in non-Middle Eastern countries [1]. For instance, in the United States, a survey of 152 universities indicated that the current rate of waterpipe use was 8.4% and ever using waterpipe was 30.5% [4].

In Saudi Arabia, waterpipe smoking is very common among adolescents and college students although studies have shown a large variation in the incidence of waterpipe use. The overall high school student smoking rate was 21.7%; and the proportion of all smokers using just waterpipe was 37.4%. In addition, 16.5% of smokers used both waterpipe and cigarettes [5]. A recent Saudi Arabian national survey indicated that overall prevalence of current tobacco smoking was 12.2% among those 15 years or older. Daily waterpipe smoking occurred in 4.3% of the total population and 1.4% current daily smokers of cigarettes, cigar, and waterpipe [6]. In the last two decades, waterpipe smoking has increased among adolescents and young adults [7] in a way that is alarming to public health officials [1].

To attract users, waterpipe tobacco is moist and flavored with many fruit flavors, but this does not make it safe to the smokers’ health. In fact, the toxicants in waterpipe tobacco/smoke are similar to those found in cigarettes [8]. The American Lung Association [9] has called waterpipe tobacco smoking “an emerging deadly trend”. In the last three decades, more evidence about health effects of waterpipe use has appeared in the literature. Studies have shown that waterpipe smoking is one of the risk factors for several tobacco-related health problems including lung cancer, cardiovascular diseases, periodontal disease, and low birth weight. Waterpipe use may lead to lung cancer [10] and smoking waterpipe has acute physiological effects on smokers which contradicted the claim that waterpipe use might be a safe alternative to cigarette smoking [11]. Waterpipe smoking is associated with markers of atherosclerosis which might increase the risk of coronary heart disease [12]. Waterpipe use may also negatively affect pregnancy outcomes. Mothers who smoked waterpipe more than one time a day were 2.4 times more likely to have a low birth weight baby compared to non-smoking mothers [13].

Waterpipe and cigarette smoking have been found to be associated with each other as cigarette smokers were four times more likely to be waterpipe users than non-cigarette smokers and waterpipe smokers were likely to be former daily cigarette users [14]. In this study, Theory of Planned Behavior (TPB) was used as a framework to predict being a cigarette smokers among current waterpipe users.

According to the TPB, the behavior is a result of the intention to do the behavior and the perceived behavioral control of the behavior. The intention can be predicted by the attitude towards behavior, the subjective norm regarding the behavior, and the perceived behavioral control over doing the behavior. The relationship between intention and actual behavior is not perfect but intention can act as a proxy for the actual behavior.

The purpose of this study was to predict being a cigarette smoker among current waterpipe users in Riyadh, Saudi Arabia using theory of planned behavior constructs. The study examined how attitude, subjective norm, and perceived behavioral control could predict intentions to smoking cigarettes and actual cigarette smoking behavior. In the last 30 years, the TPB has been one of the dominant theories used in understanding health-related behavior [15]. The TPB has been used successfully in predicting and understanding a number of health-related behaviors including tobacco smoking [16], alcohol consumption, and substance use [17].

## Methods

### Participants

This study was part of a larger study of 622 waterpipe users recruited in 2015 from 15 waterpipe lounges in Riyadh, Saudi Arabia. Only the 406 waterpipe smokers who reported started smoking waterpipe before cigarettes were included in this study. Additionally, they were males, age of 18 years or older, and had the ability to use an electronic device as the survey was taken via iPad tablets. Institutional Review Board approval from Loma Linda University was obtained before beginning data collection and informed consent was required from each participant before taking the survey. There was a total of 169 participants who were contacted to participate in the study, but refused. For the study participants, each survey question had to be answered to move to the next one; therefore, no missing data were expected.

### Procedures

The original questionnaire was translated from English into Arabic using Brislin’s model [18, 19]. According to that model, two bilingual individuals fluent in English and Arabic were needed. One bilingual expert translated the original questionnaire from the source language (English) into the participants’ language (Arabic), and then the second bilingual expert back-translated the Arabic into English without having access to the original English version. Both versions were compared, then the first translator revised the Arabic and the second translator again translated the revised Arabic back into English. This process was repeated until no variation was found in the meaning between the two versions.

A list of all waterpipe lounges in Riyadh, Saudi Arabia were obtained from the Riyadh Municipality, Department of Environmental Health. From the 70 waterpipe lounges listed, 15 waterpipe lounges were selected randomly using Microsoft Excel. Usually, each waterpipe lounge contains small sections with a number assigned to it. Each section is separated from other sections to be convenient for the customer’s privacy. In order to randomize the sample in each waterpipe lounge, the section numbers were sorted into random order using Excel. Then, starting from the top of the list, each participant(s) in a section was contacted. This process continued until at least 40 waterpipe smokers were recruited from each lounge. This allowed a total of at least 600 participants from the 15 selected waterpipe lounges.

Upon giving informed consent, participants completed a survey on iPad tables including sample demographics (age, marital status, income, work status & education), tobacco withdrawal symptoms, and smoking history (waterpipe and cigarette). The survey was developed using the Qualtrics Online Survey Software and administered by the investigator to the participants using the Qualtrics Online Survey App for the iPad tablet (Qualtrics Labs, Inc. © 2015, version, Jan., 2015).

The dependent variables included in this study were intention (toward cigarette smoking) and current cigarette smoking behavior. The National Survey on Drug Use and Health defines current cigarette use as having smoked part or all of a cigarette during the last 30 days [20]. Thus, to assess current cigarette smoking status participants were asked “Have you smoked even one cigarette in the last 30 days?” Those who were current cigarette smokers were also asked whether they initially started smoking waterpipe or cigarettes. Only those who initially started smoking waterpipe were included in these analyses.

The main independent variables were the TPB constructs—attitudes, subjective norms, perceived behavioral control, and intention. The scaling procedure to create questions for attitude, subjective norm, and perceived behavioral control, as well as intention was based on a modified version of the theory of planned behavior questionnaire developed by Icek Ajzen [21].

Theory of Planned Behavior questions in this study included the following:

*Intention toward Cigarette Smoking.* Four questions assessed behavioral intentions. Participants were asked questions using a 7-point scale (from 1 to 7 where 1 = *strongly disagree/ extremely unlikely* and 7 = *strongly agree/extremely likely*). For example, participants answered questions such as: I intend to smoke cigarettes when waterpipe is not available *(Strongly Agree/Strongly Disagree)*.

How likely is it that you will smoke cigarettes when you do not have time to prepare and smoke waterpipe? *(Extremely likely/Extremely unlikely)*.

*Attitude toward Cigarette Smoking.* Five questions were included to assess participants’ attitude toward smoking cigarettes. A set of semantic differential scales were used with two bipolar adjectives on 7-point evaluative scales. Scales of both instrumental (e.g., *valuable*—*worthless*) and experiential (e.g., *pleasant*—*unpleasant*) types were included such as:

To me to smoke cigarettes when I do not have time for waterpipe smoking is (*Pleasant/Unpleasant*).

*Subjective Norm.* Subjective norm was assessed with five questions used to assess participants’ perception of what other people think about engaging in cigarette smoking in addition to waterpipe smoking. The items included both injunctive and descriptive items. For example, a 7-point scale (1 = *strongly disagree* & 7 = *strongly agree*) was used as follows:

*Injunctive.*

Most people who are important to me think that (*I should/I should not*) smoke cigarettes as a substitute for waterpipe smoking when a waterpipe is not available.

*Descriptive.*

The people in my life whose opinions I value *(Smoke/do not smoke)* cigarettes when waterpipe is unavailable.

*Perceived behavioral control*. Five questions about perceived behavioral control were used to capture participants’ confidence that they were able to perform cigarette smoking behavior. There were two types of items, those that assessed capability and those that assessed controllability. For example:

*Capability.*

For me to smoke cigarette when I do not have time for waterpipe would be *(Impossible/Possible).*

*Controllability.*

How much control do you believe you have over not smoking cigarettes no matter what the situation is? *(No control/complete control).*

Additional independent variables used in the study included waterpipe use, tobacco withdrawal symptoms, smoking history and demographics. These are described below.

*Waterpipe use.* Since all participants were recruited from waterpipe lounges while smoking, all participants were current waterpipe users. Frequency of waterpipe use was assessed by the number of bowls (heads) of waterpipe tobacco consumed per week. The years of waterpipe use was defined by the number of years since waterpipe smoking initiation.

*Tobacco Withdrawal Symptoms.* Tobacco withdrawal symptoms were measured by using a modified version of the Hooked on Nicotine Checklist [21]. This measure was developed for nicotine addiction and primarily for use with cigarettes, so the word “cigarette” was replaced by “waterpipe” and the context was modified to be relevant to waterpipe smoking characteristics. The measure contains 10 *yes* and *no* questions scored as the number of *yes* responses. A zero score (answering *no* to all ten questions) means a person enjoys full autonomy over tobacco use. Any score above zero reveals that a person has lost full autonomy over tobacco use. As the score goes up, the more autonomy is lost. An example item from this scale is:

*Do you ever have strong cravings to smoke waterpipe?*

*Smoking History and Demographics.* Five questions were used to evaluate the participants smoking history: age of initiation of waterpipe use, ever smoked cigarettes, age of initiation of cigarette use, number of cigarettes smoked in a week (if they were cigarette users), and whether the participants initially started smoking waterpipe or cigarettes. In addition, five questions were used to evaluate the sample demographics of age, marital status, education level, work status, and income.

### Statistical analyses

The statistical analyses were implemented with IBM SPSS Statistics (Version 23; IBM Corporation 1989, 2015). Descriptive statistics were presented as means and standard deviation (*SD*) or numbers (*n*) and percentages (*%*) as appropriate. Logistic regression models were used to examine the association of TPB constructs with current cigarette use among current waterpipe users. Multiple linear regression was used to examine the association of smoking behavioral intention with attitude, subjective norm, and perceived behavioral control.

## Results

Of the 406 participants, 70.9% were exclusive waterpipe smokers and 29.1% smoked both waterpipe and cigarettes. The sample mean age was 26.97 (*S.D.* 6.82); however, the exclusive waterpipe users mean age, 27.82 (*S.D.* 7.02), was significantly higher than the mean age of those who used both waterpipe and cigarettes 24.89 (*S.D.* 5.82), *t* (404) = 4.01, *p* < .001. Sample characteristics in Table 1 show also that the mean of waterpipe initiation age was under 21 years of age. Because our sample was relatively young, the mean years of waterpipe use was six years. Our data show that the mean duration of a waterpipe smoking session was 88.38 min and the largest group of participants were single, college or post graduate, or government employees. Those who smoked only waterpipe had more income compared with those who smoked both cigarettes and waterpipe. In addition, less educated participants appeared to use both cigarettes and waterpipe whereas educated participants smoked waterpipe only. Nevertheless, government employees seemed to smoke waterpipe only; whereas waterpipe and cigarette users were likely to be students.

**Table 1:** Sample characteristics (*n* = 406) for waterpipe users in Riyadh, Saudi Arabia, 2015

	Total	Exclusive Waterpipe Users (*n* = 288)	Waterpipe & Cigarettes Users (*n* = 118)	
	*Mean ± SD or n (%)*	*Mean ± SD or n (%)*	*Mean ± SD or n (%)*	*p*-value
Age	26.97 ± 6.82	27.82 ± 7.02	24.89 ± 5.82	*< .001*
Waterpipe initiation age	20.99 ± 4.49	21.72 ± 4.71	19.20 ± 3.30	*<.001*
Cigarettes initiation age	19.72 ± 4.20	NA	19.72 ± 4.20	*NA*
No. of cigarettes smoked/week	22.21 ± 39.18	NA	22.21 ± 39.18	*NA*
Years of waterpipe use	5.98 ± 5.35	6.10 ± 5.44	5.69 ± 5.13	*.483*
Waterpipe frequency use/week	7.62 ± 6.92	7.70 ± 6.71	7.45 ± 7.45	*.742*
Waterpipe session duration (min)^a^	88.38 ± 45.34	86.67 ± 44.88	92.56 ± 46.39	*.238*
*TPB Constructs*				
Attitude	1.98 ± 1.12	1.72 ± .97	2.60 ± 1.21	*< .001*
Subjective Norms	3.05 ± 1.17	2.92 ± 1.14	3.38 ± 1.19	*< .001*
Perceived Control	3.67 ± 1.13	3.32 ± .94	4.51 ± 1.10	*< .001*
Intentions	2.22 ± 1.69	1.58 ± 1.07	3.79 ± 1.87	*< .001*
*Marital Status*				
Single	267 (65.8%)	170 (59.0%)	97 (82.2%)	*< .001*
Others	139 (34.2%)	118 (41.0%)	21 (17.8%)	
*Education Level*				
High school or less	194 (47.8%)	127 (44.1%)	67 (56.8%)	*.020*
College/ post graduate	212 (52.2%)	161 (55.9%)	51 (43.2%)	
*Work Status*				
Government employee	139 (34.2%)	108 (37.5%)	31 (26.3%)	*.002*
Non-government employee	101 (24.9%)	80 (27.8%)	21 (17.8%)	
Self-employed	22 (5.4%)	15 (5.2%)	7 (5.9%)	
Student	125 (30.8%)	75 (26.0%)	50 (42.4%)	
Unemployed	19 (4.7%)	10 (3.5%)	9 (7.6%)	
*Income/month*				
SR 3000 or Less	133 (32.8%)	76 (26.4%)	57 (48.3%)	*< .001*
SR 3001 to 6000	70 (17.2%)	48 (16.7%)	22 (18.6%)	
SR 6001 to 9000	75 (18.5%)	55 (19.1%)	20 (16.9%)	
More than SR 9000	128 (31.5%)	109 (37.8%)	19 (16.1%)	
*Exercise*				
Never	117 (28.8%)	73 (25.3%)	44 (37.3%)	*.095*
1 to 2 times per week	184 (45.3%)	135 (46.9%)	49 (41.5%)	
3 to 4 times per week	62 (15.3%)	46 (16.0%)	16 (13.6%)	
5 or more times per week	43 (10.6%)	34 (11.8%)	9 (7.6%)	

Table 2 illustrates the association between TPB constructs and background variables. The table represents simple Pearson product moment correlations between the TPB variables and other study variables. The higher scores on the TPB constructs represent more favorable attitude, subjective norm, perceived behavioral control, and intention toward using cigarettes. Those with attitude, subjective norm, perceived behavioral control, and intentions favorable toward cigarette use tended to be younger and to have started waterpipe at younger age. In addition, tobacco withdrawal symptoms had significant positive association with all theory constructs. The more favorable of attitude, subjective norm, perceived behavioral control, and intentions toward cigarette smoking were, the higher tobacco withdrawal symptoms score was. Possibly, since those who are more favorable to cigarette smoking are also more likely to be cigarette smokers, it may well be that those more favorable have higher withdrawal symptoms because they are getting more nicotine and, hence, are more addicted.

**Table 2:** Associations between TPB’s constructs and background variables (*n* = 406) for waterpipe users in Riyadh, Saudi Arabia, 2015

	Favorable toward cigarette smoking			
	Attitude	Subjective Norm	Perceived Control	Intentions
Pearson Correlation				
Age	−0.19*	−0.20*	−0.17*	−0.20*
Waterpipe initiation age	−0.12*	−0.14*	−0.16*	−0.23*
Withdrawal symptoms scores	0.13*	0.21*	0.14*	0.22*
No. of cigarettes smoked/week	0.10	0.03	0.19	0.40*
Waterpipe session duration(min)	0.09	0.06	−0.02	−0.02
Waterpipe frequency use/week	−.01	.07	−.00	.06
Years of cigarettes use	−0.18*	−0.07	−0.13	−0.08
Years of waterpipe use	−0.14*	−0.14*	−0.08	−0.06

The Pearson product moment correlations, the * represents significance at an alpha of 0.05

Favorable attitude for cigarette use was significantly associated with less years of cigarette use. In other words, as years of cigarettes use increased, there would be less favorable attitude toward cigarettes. Furthermore, strong association was found between number of years waterpipe use and number of years cigarette use *r*(*404*) = .67, *p* = <.0005. Even when age was controlled for, there was a significant association of years of use of both waterpipe and cigarettes *r*(*404*) = .28, *p* = <.0005. In addition, favorable attitude and subjective norm toward cigarette use were associated with less years of waterpipe use. Strong association was found between intentions and the number of cigarettes smoked per week. Higher favorable intentions toward cigarettes use was associated with more cigarettes consumption per week.

Table 3 shows descriptive statistics for categorical study variables showing the means for each Theory of Planned Behavior variable within each subcategory of a categorical study variable. Participants who were single were significantly higher than other groups to have favorable attitude, subjective norm, perceived behavioral control, and intentions toward cigarettes. Education levels had no significant association to any TPB constructs. Furthermore, smokers with the least income had significantly higher attitude, subjective norms, perceived behavioral control, and intention scores favorable to smoke cigarettes compared to those with high income.

**Table 3:** Associations between TPB’s Constructs and other Variables (*n* = 406) for Waterpipe Smokers in Riyadh, Saudi Arabia, 2015

	Attitude (Mean ± SD)	Subjective Norm (Mean ± SD)	Perceived Control (Mean ± SD)	Intentions (Mean ± SD)
Marital status				
Single	2.11 ± 1.16_a_	3.16 ± 1.09_a_	3.79 ± 1.13_a_	2.44 ± 1.67_a_
Others	1.73 ± .99_b_	2.84 ± 1.30_b_	3.43 ± 1.08_b_	1.80 ± 1.45_b_
Education Levels				
High school or less	2.07 ± 1.14_a_	3.18 ± 1.11_a_	3.77 ± 1.18_a_	2.35 ± 1.75_a_
College & post graduate	1.89 ± 1.09_a_	2.93 ± 1.22_a_	3.57 ± 1.08_a_	2.10 ± 1.61_a_
Work Status				
Government employee	1.84 ± 1.11_a_	3.03 ± 1.24_a_	3.45 ± 1.10_a_	2.04 ± 1.56_a_
Non-government employee	1.81 ± .97_a_	2.98 ± 1.14_a_	3.58 ± 1.02_a,b_	2.02 ± 1.58_a_
Self-employed	1.97 ± 1.01_a_	2.72 ± 1.24_a_	3.93 ± 1.49_a,b_	2.75 ± 2.32_a_
Student	2.23 ± 1.21_a_	3.13 ± 1.10_a_	3.89 ± 1.12_b_	2.42 ± 1.68_a_
Unemployed	2.22 ± 1.12_a_	3.45 ± 1.18_a_	3.93 ± 1.10_a,b_	2.71 ± 2.04_a_
Monthly Income				
SR 3000 or Less	2.21 ± 1.20_a_	3.21 ± 1.11_a_	3.95 ± 1.12_a_	2.51 ± 1.78_a_
SR 3001 to 6000	1.94 ± 1.02_a,b_	3.29 ± 1.12_a_	3.54 ± 1.17_a,b_	2.36 ± 1.80_a,b_
SR 6001 to 9000	1.89 ± 1.07_a,b_	3.11 ± 1.12_a,b_	3.71 ± 1.08_a,b_	2.14 ± 1.65_a,b_
More than SR 9000	1.81 ± 1.08_b_	2.73 ± 1.24_b_	3.42 ± 1.08_b_	1.90 ± 1.48_b_
Exercise				
Never	2.04 ± 1.10_a_	3.00 ± 1.16_a_	3.84 ± 1.22_a_	2.64 ± 1.97_a_
1 to 2 times per week	2.06 ± 1.16_a_	3.05 ± 1.15_a_	3.68 ± 1.07_a,b_	2.10 ± 1.57_b_
3 to 4 times per week	1.82 ± 1.01_a_	3.02 ± 1.15_a_	3.59 ± 1.08_a,b_	2.24 ± 1.62_a,b_
5 or more times per week	1.68 ± 1.07_a_	3.24 ± 1.35_a_	3.25 ± 1.04_b_	1.56 ± 1.03_b_

For the means, values within each column not sharing the same subscript are significantly different at an alpha .05

SR = Saudi Riyal ($1 = SR 3.75).

### Dimension of TPB constructs in association with current cigarettes use

Logistic regression analysis was performed with the TPB constructs to predict being a current cigarette smoker. In Table 4, all TBP constructs were added to the model and all of them predicted current cigarette smoking except subjective norm. Moreover, multiple linear regression in Table 5 shows that intention was predicted by attitude and perceived behavioral control but not subjective norm.

**Table 4:** Logistic regression of whether smoking cigarettes on TPB constructs (*n* = 406) for waterpipe users in Riyadh, Saudi Arabi, 2015

	B	Sig.	OR	95% C.I. for OR	
				Lower	Upper
Attitude	.451	.007	1.571	1.133	2.177
Subjective Norm	−.007	.967	.993	.722	1.367
Perceived Control	.821	.000	2.274	1.509	3.426
Intention	.567	.000	1.764	1.406	2.213
Constant	−7.433	.000	.001

**Table 5:** MLR of intentions on attitude, subjective norm, & perceived control (*n* = 406) for waterpipe users in Riyadh, Saudi Arabi, 2015

	B	Sig.	OR	95.0% C.I. for B	
				Lower	Upper
Model (Constant)	−1.489	.238	.000	−1.957	−1.022
Attitude	.515	.063	.000	.391	.638
Subjective Norm	.053	.058	.367	−.062	.167
Perceived Control	.691	.059	.000	.574	.807

To dig deeper into the relationship between TPB constructs, simple linear regression was used to scrutinize the relationship of intention with attitude, subjective norm, and perceived behavioral control in separate analyses. Each one, by itself, strongly predicted intention (Attitude: *B* = .780, *C.I* [.654, .907] *p =* <0.0005), (Subjective norm: *B* = .431, *C.I* [.296, .565] *p =* <0.0005), and (Perceived behavioral control: *B* = .883, *C.I* [.765, 1.002] *p =* <0.0005). This suggested that the prediction of intention by subjective norm was mediated by the other TPB constructs. When we controlled for perceived behavioral control, subjective norm was still a strong predictor for the intention (Subjective norm: *B* = .216, *C.I* [.099, .332] *p = <*0.0001). In addition, when we controlled for attitude, subjective norm was also a predictor for the behavior but was not as strong as it had been after controlling for perceived behavioral control (Subjective norm: *B* = .157, *C.I* [.026, .288] *p =* 0.019).

Additional analysis using Hayes’ PROCESS macro [22] revealed that there was no direct effect of subjective norm on the intentions (*B* = 0.053, 95% CI [−.062, 0.167], *p* = 0.367), yet subjective norm had a statistically significant indirect effect on the intentions through both attitude and perceived behavioral control— attitude: *B* = 0.197, 95% CI [0.139, 0.269]; Perceived behavioral control: *B* = 0.181, 95% CI [0.113, 0.260].

## Discussion

In this study, the TPB was applied to predict behavioral intention and cigarette smoking behavior among current waterpipe users in Riyadh, Saudi Arabia. To our knowledge, this is the first study that utilized a theory-based approach to understand cigarette smoking intention and cigarette smoking behavior among waterpipe users. The study provided support for the utility of the TPB to predict cigarette smoking behavior among current waterpipe users, although the subjective norm to smoke cigarettes was not an essential element for that prediction. This may be due to the fact that all participants were current waterpipe smokers, it might be that a person’s beliefs about whether friends and people of importance to him had less influence or pressure to engage or not to engage in cigarette smoking because a person was already involved in one type of smoking behavior— habitual waterpipe tobacco use. In addition, previous research suggested that subjective norm was more likely to be a predictor of smoking behavior among adolescents, whereas attitudes were more likely to be a predictor of smoking behavior among adults [23]. As our sample study were adults and they have already engaged in one smoking behavior— waterpipe— subjective norm was not an important direct predictor for intentions toward cigarettes or actual cigarettes use.

Behavioral intention to smoke cigarettes could be predicted by the TPB constructs. The results indicated that for current waterpipe users, attitude (regardless of negative attitude for cigarettes) toward cigarette smoking and perception of the ease and difficulty of smoking cigarettes directly influenced intention to use cigarettes, explaining 46% of the variance in cigarette smoking intention—*F (3405), p < 0.001*. Attitude and perceived behavioral control were the strong predictors of the intentions, whereas subjective norm had no direct influence in the intention to smoke cigarettes. This is consistent with some studies that utilized the TPB which found that subjective norms were either the weakest predictor of intentions [24–26], or were non-significant in the prediction of intention [27, 28].

Our study also showed that those who used both waterpipe and cigarettes were significantly higher in all TPB constructs than those who were exclusive waterpipe users. This is logical since they did smoke cigarettes, they would have favorable attitude towards cigarettes, preconception about self and others of smoking cigarettes, and strong beliefs of their ability to smoke cigarettes; however, this may raise a question about why those who smoked both waterpipe and cigarettes had more perceived behavioral control of cigarette smoking than exclusive waterpipe smokers. One explanation is that since they had experienced smoking cigarettes and knew its negative influences, their feelings of control over not smoking cigarettes could be high due to currently smoking waterpipe. Waterpipe could be acting as a substitute for cigarettes which degraded the craving for cigarettes and provided them with more control. Exclusive waterpipe users seemed to have less predisposition to smoke cigarette compared to those who were not exclusive waterpipe users which was expected. Current waterpipe users with cigarette smoking or past cigarette smoking history had more favorable attitude, higher social approval, and higher self-beliefs of smoking cigarettes than those who never smoked cigarettes.

The findings of this study could be useful in prevention/intervention programs that are intended to prevent or reduce tobacco smoking behaviors. Prevention and intervention programs might be directed to the attitude and perceived behavioral control by targeting underlying behavioral and control beliefs. Though additional research to enumerate what these beliefs are would be useful.

One of the limitations of this study design is lack of the ability to determine causal relationships; our results are only correlational. Also this study was based on self-reported responses which might be susceptible to recall bias. In addition, the results of this study may not be generalized to female waterpipe smokers as the study sample included only males but it could benefit other geographical areas that share similar cultural backgrounds.

Regardless of these limitations, this study has some advantages. The study went through two levels of randomization, one at the level of the waterpipe lounges and another one within the sections of each waterpipe lounge. The randomization process may reduce the potential bias and the sample was likely to be highly representative of the population that was studied. Another advantage of this study was the electronic approach used to collect data. The iPad tablets with Internet access which were used to input and store data electronically not only saved paper, but sped up the process, produced cleaner data, and reduced chances of error in transcribing responses since no transcribing was necessary.

## Conclusion

The study indicated that the TPB successfully predicted cigarette smoking behavior among waterpipe users. The TPB could provide solid explanations of intention to start cigarette use. Subjective norm was not a predictor of the intention but it had indirect effects on intention through attitude and perceived behavioral control. These findings might usefully be integrated in an intervention program aimed at reducing or preventing smoking initiation, especially among young adults since our sample was relatively young. Further investigation to understand the mutual relationship between waterpipe and cigarette use is needed to establish an effective approach to curb enthusiasm for smoking tobacco.

## Abbreviations

SR: Saudi Riyal
TPB: Theory of Planned Behavior
